# Supplementary material for: Pyruvate Dehydrogenase Kinase 1 inhibition mediated oxidative phosphorylation enhancement in cartilage promotes osteoarthritis progression
Source: BMC Musculoskelet Disord. 2023 Jul 20;24:597. doi: 10.1186/s12891-023-06585-6 (PMC10357736; doi:10.1186/s12891-023-06585-6)
Supplement: Supplementary file 1 — Additional file 1. [file 12891_2023_6585_MOESM1_ESM.zip › MF.Gsea.1653627316242/GOMF_HISTONE_ACETYLTRANSFERASE_BINDING.html]

Details for gene set GOMF\_HISTONE\_ACETYLTRANSFERASE\_BINDING[GSEA]

|  || Dataset | 55457\_collapsed\_to\_symbols.cls.cls #OA\_versus\_normal.cls.cls #OA\_versus\_normal\_repos |
| Phenotype | cls.cls#OA\_versus\_normal\_repos |
| Upregulated in class | OA |
| GeneSet | GOMF\_HISTONE\_ACETYLTRANSFERASE\_BINDING |
| Enrichment Score (ES) | 0.35273433 |
| Normalized Enrichment Score (NES) | 1.1463916 |
| Nominal p-value | 0.2733813 |
| FDR q-value | 0.6155169 |
| FWER p-Value | 1.0 |
Table: GSEA Results Summary

  

Fig 1: Enrichment plot: GOMF\_HISTONE\_ACETYLTRANSFERASE\_BINDING      
 Profile of the Running ES Score & Positions of GeneSet Members on the Rank Ordered List

  

| SYMBOL | TITLE | RANK IN GENE LIST | RANK METRIC SCORE | RUNNING ES | CORE ENRICHMENT || 1 | TRIM68 | tripartite motif containing 68 [Source:HGNC Symbol;Acc:HGNC:21161] | 34 | 1.023 | 0.1216 | Yes |
| 2 | PAX6 | paired box 6 [Source:HGNC Symbol;Acc:HGNC:8620] | 304 | 0.746 | 0.1913 | Yes |
| 3 | EID1 | EP300 interacting inhibitor of differentiation 1 [Source:HGNC Symbol;Acc:HGNC:1191] | 456 | 0.679 | 0.2620 | Yes |
| 4 | ECD | ecdysoneless cell cycle regulator [Source:HGNC Symbol;Acc:HGNC:17029] | 682 | 0.598 | 0.3171 | Yes |
| 5 | BCAS3 | BCAS3 microtubule associated cell migration factor [Source:HGNC Symbol;Acc:HGNC:14347] | 1471 | 0.433 | 0.3085 | Yes |
| 6 | PCNA | proliferating cell nuclear antigen [Source:HGNC Symbol;Acc:HGNC:8729] | 1557 | 0.419 | 0.3527 | Yes |
| 7 | GLI3 | GLI family zinc finger 3 [Source:HGNC Symbol;Acc:HGNC:4319] | 2861 | 0.264 | 0.2837 | No |
| 8 | TAF7 | TATA-box binding protein associated factor 7 [Source:HGNC Symbol;Acc:HGNC:11541] | 3035 | 0.246 | 0.3001 | No |
| 9 | TRIP4 | thyroid hormone receptor interactor 4 [Source:HGNC Symbol;Acc:HGNC:12310] | 4105 | 0.146 | 0.2348 | No |
| 10 | STAT1 | signal transducer and activator of transcription 1 [Source:HGNC Symbol;Acc:HGNC:11362] | 4495 | 0.113 | 0.2183 | No |
| 11 | TP53 | tumor protein p53 [Source:HGNC Symbol;Acc:HGNC:11998] | 5511 | 0.032 | 0.1433 | No |
| 12 | MEF2A | myocyte enhancer factor 2A [Source:HGNC Symbol;Acc:HGNC:6993] | 5628 | 0.022 | 0.1370 | No |
| 13 | SIRT2 | sirtuin 2 [Source:HGNC Symbol;Acc:HGNC:10886] | 6279 | -0.026 | 0.0896 | No |
| 14 | FOXP3 | forkhead box P3 [Source:HGNC Symbol;Acc:HGNC:6106] | 7035 | -0.082 | 0.0410 | No |
| 15 | CITED2 | Cbp/p300 interacting transactivator with Glu/Asp rich carboxy-terminal domain 2 [Source:HGNC Symbol;Acc:HGNC:1987] | 7100 | -0.087 | 0.0467 | No |
| 16 | KANSL1L | KAT8 regulatory NSL complex subunit 1 like [Source:HGNC Symbol;Acc:HGNC:26310] | 7451 | -0.114 | 0.0334 | No |
| 17 | MTF1 | metal regulatory transcription factor 1 [Source:HGNC Symbol;Acc:HGNC:7428] | 10125 | -0.334 | -0.1336 | No |
| 18 | ZBTB7A | zinc finger and BTB domain containing 7A [Source:HGNC Symbol;Acc:HGNC:18078] | 11600 | -0.516 | -0.1855 | No |
| 19 | EGR1 | early growth response 1 [Source:HGNC Symbol;Acc:HGNC:3238] | 12052 | -0.607 | -0.1468 | No |
| 20 | SP1 | Sp1 transcription factor [Source:HGNC Symbol;Acc:HGNC:11205] | 12557 | -0.777 | -0.0916 | No |
| 21 | CEBPB | CCAAT enhancer binding protein beta [Source:HGNC Symbol;Acc:HGNC:1834] | 12804 | -0.973 | 0.0075 | No |
Table: GSEA details [plain text format]

  

Fig 2: GOMF\_HISTONE\_ACETYLTRANSFERASE\_BINDING      
 Blue-Pink O' Gram in the Space of the Analyzed GeneSet

  

Fig 3: GOMF\_HISTONE\_ACETYLTRANSFERASE\_BINDING: Random ES distribution      
 Gene set null distribution of ES for **GOMF\_HISTONE\_ACETYLTRANSFERASE\_BINDING**
